# Nasal MRSA carriage is a risk factor for development of antibiotic resistance in diabetic foot ulcers and is significantly higher than diabetic and non-diabetic individuals without foot ulcer

**DOI:** 10.1186/s12879-023-08673-3

**Published:** 2023-10-26

**Authors:** Poulami Mukherjee, Shouvik Paul, Tanmoy Dutta, Shankha Nath, Bikramaditya Ghosh, Debika Chatterjee, Satinath Mukhopadhyay, Souvik Mukherjee

**Affiliations:** 1 Human Microbiome Research Laboratory, National Institute of Biomedical Genomics (NIBMG), Kalyani, West Bengal India; 2 Present address: Ramakrishna Mission Seva Pratishthan/ Vivekananda Institute of Medical Sciences, Kolkata, West Bengal India; 3 Regional Centre for Biotechnology (RCB), Faridabad, Haryana India; 4 Chemical Examination Laboratory, Govt of West Bengal, Kolkata, West Bengal India; 5 Dept. of Endocrinology and Metabolism, IPGME&R, Kolkata, West Bengal India

**Keywords:** Diabetic foot ulcer, *Staphylococcus aureus*, MRSA carriage, Multiplex PCR, HbA1C

## Abstract

**Background:**

Diabetic foot ulcer (DFU) is a major complication of diabetes often impacted by polymicrobial infection in the wound site. Diabetic patients are immunocompromised in nature and hence vulnerable to infection once the skin barrier is breached. Microbiological culture-based methods show that *Staphylococcus aureus* (SA) is the most frequently isolated bacteria from the DFU wounds. SA and its most clinically important antibiotic resistant variant methicillin-resistant *S. aureus* (MRSA) are commonly found in the nasal vestibule and colonization of SA as well as MRSA in any wound site can aggravate the condition. We hypothesize that the presence of nasal MRSA carriage can serve as a potential risk factor contributing to the emergence of antibiotic resistance in diabetic foot ulcer wounds.

**Methods:**

In the present study, we have compared the carriage of SA and MRSA in nasal cavity and foot skin among DFU patients (D+F+, *n* = 50), diabetic patients without any ulcer (D+F-, *n* = 50), and healthy controls (D-F-, *n* = 40) by using bacterial culture and PCR based methods. The D+F+, D+F- and D-F-individuals were further categorized based on the presence or absence of MRSA and clinical parameters were compared between MRSA+ ve and MRSA-ve individuals in each of the three groups mentioned above.

**Results:**

Our results show that, (a) nasal MRSA carriage is significantly higher (*p* < 0.05) in D+F+ group than the D+F- and D-F- and significantly associated with wound MRSA carriage in D+ F+ individuals (O.R. = 4.09; 95% C.I. = 1.12–15.05) and (b) the HbA1C level is significantly higher (*p* < 0.02) in wound MRSA positive, compared to MRSA negative D+F+ patients. Interestingly more than half of the MRSA (64%) isolated from DFU wound were identified to be multidrug resistant.

**Conclusion:**

These findings strongly suggest that nasal MRSA carriage can act as a risk factor for development of antibiotic resistance in diabetic foot ulcers and it is therefore important to screen nasal and wound sites of these patients regularly. We have also developed a rapid multiplex PCR assay to detect MRSA from clinical isolates or microbial DNA isolated from clinical samples in the hospital settings.

## Background

Diabetes is a chronic metabolic disease characterized by elevated levels of blood glucose that affects almost 537 million people worldwide and 71 million people in India [1]. Diabetic foot ulcer (DFU) is a major complication of diabetes whose pathogenesis is poorly understood. An estimated, 25% of diabetic patients have the risk of developing DFU during their lifetime [2]. Even after standard care and treatment 30% of the DFU patients cannot recover from the ulcer and eventually need to amputate their lower limbs [3]. The previous studies show that DFUs are often impacted by polymicrobial infection in the wound site and *S. aureus* (SA) is the most commonly isolated bacteria from both infected and non-infected DFU patients [4–6]. It is often difficult to determine whether SA can act as a common colonizer or primary pathogen [7]. SA colonizes in multiple body sites, but it is found most frequently in the anterior nares and nasal vestibule. SA is a major public health concern because of its increasing virulence and resistance to a broad spectrum of antibiotics [8–13]. Studies have shown that methicillin resistant *Staphylococcus aureus* (MRSA) worsens the ulcer and increases the chances of treatment failures that leads to osteomyelitis mostly requiring amputation of the lower limb [14]. The mortality rate of MRSA+ ve DFU patients is higher than MRSA-ve DFU patients and the prevalence of MRSA in DFU is increasing at an alarming rate worldwide [15]. MRSA contains a special mobile genetic element Staphylococcal cassette chromosome mec (SCCmec), that confers resistance to a wide range of beta-lactam antibiotics including methicillin [16]. DFU patients are known to be immunocompromised and are susceptible to pathogens. The carriage of SA and MRSA in DFU wounds can act as a risk factor by worsening the disease progression and delaying the wound healing. [17–20]. Nasal cavity being one of the potential reservoirs of SA, we hypothesize that DFU individuals with high nasal SA and/or MRSA carriage are at significantly higher risk of carrying SA and/or MRSA in their foot ulcer wounds. The primary objective of our study is to identify the role of nasal SA and MRSA carriage in the development of antibiotic resistance in the foot ulcer wounds of DFU patients. The antibiotic resistance in the Diabetic patients is known to be higher due to their immunocompromised condition. Hence, we have also compared the nasal and foot skin carriage of MRSA in DFU patients (D+F+) with that of the Diabetic patients (D+F-) without foot ulcer to identify if the Diabetic patients act as reservoirs of MRSA in their nasal and foot skin sites. Thus, our study provides novel insight on the reduction in antibiotic resistance in DFU wounds by management of nasal MRSA carriage in DFU patients. The management of the nasal MRSA carriage in Diabetic patients without foot ulcers may also reduce the chance of development of antibiotic resistant DFU wounds in these patients in the future. This will further expedite wound healing in these patients.

## Methods

### Study design and patient recruitment

DFU and diabetic patients without foot ulcer were included in this study from Diabetic Foot Clinic under the Diabetes Outpatients Services of IPGME&R Kolkata, India. The DFU patients were included after stringent inclusion–exclusion criteria, i.e., chronic, infected, and deep ulcers [21]. These ulcers were categorized as grade 2 in severity according to both the University of Texas (Grade 2; stages B- Infection and D- Infection & Ischemia) and IWGDF/IDSA (International Working Group on the Diabetic Foot/Infectious Disease Society of America) systems [21, 22]. Grade 2 ulcers involve localized skin and subcutaneous tissue infection with erythema exceeding 2 cm showing signs of inflammation or affecting deeper structures such as tendons without bone involvement and these patients did not exhibit systemic inflammatory response signs. Additionally, we confirmed the presence of inflammation by observing a temperature difference of at least 2 degrees Celsius between the ulcer site and the corresponding area on the opposite foot. DFU wounds in Indian patients are found to be more infectious and severe than in the Western populations [23]. Healthy individuals are mostly either the unrelated accompanying person of the patients or hospital staff. In this study, three groups were present: chronic diabetic foot ulcer patients (D+F+, *n* = 50), individuals with diabetes but without any history of foot ulcer (D+F-, *n* = 50) and healthy individuals without any history of diabetic foot ulcer (D-F-, *n* = 40). Samples were collected from these three groups only if they did not take any antibiotics for the last 2 weeks. Most of the Diabetic Foot Clinic patients had neuropathic DFU. Those with systemic inflammation are on antibiotics, were excluded from our study. We enrolled DFU patients with localized foot infection only who weren't on antibiotics. Swab samples were collected from all three groups (D+F+, D+F-, D-F-) by using Levine’s technique and transported in sterile tube along with autoclaved 1 × phosphate buffered saline (PBS) and processed immediately. Swab samples were collected from different body sites of study participants. For the D+F+ group, swabs were collected from three sites (the wound site of the foot, intact skin site on the opposite foot, and the nasal site), while for the D+F- and D-F- groups, swabs were collected from two sites (the nasal site and plantar foot site).

Fasting blood glucose (FBG), Post Prandial blood glucose (PPBG) and the HbA1C levels were checked for all the participants (D+F+, D+F-, D-F-) included in this study. D+F+ and D+F- patients showed no significant differences in clinical parameters (Student’s T-test). To rule out the possibility of individuals from the D+F- group becoming a D+F+ patient in near future, we have included only those D+F- patients whose duration of diabetes was significantly higher (*p* < 0.05) than the D+F+ group. Patient recruitment and collection of medical and clinical data was performed by experienced clinicians from the Diabetic Foot OPD of IPGME&R hospital. They were responsible for conducting the assessments, recording the measurements, and providing the necessary medical and clinical information for the study.

### Microbiological processing

Swab samples were cultured in Mannitol Salt Agar (HiMedia; SPH118) plates (MSA plates) for selective isolation of *Staphylococcus aureus*. Isolated SA colonies were then cultured in HiCrome-Rapid MRSA Agar plates (HiMedia; M1974) for isolating methicillin resistant *Staphylococcus aureu*s (MRSA). Minimum inhibitory concentration (MIC) of cefoxitin was also checked for the *S. aureus* to detect methicillin resistance or susceptibility of all SA culture isolates. Susceptibility/resistance of SA was determined based on the latest CLSI guidelines, i.e., are called methicillin resistant if the MIC is at least 8 μg/ml; intermediate, when MIC is in between 4–8 μg/ml; and called sensitive, when MIC is less than 4 μg/ml [24].

### Microbial DNA isolation and Multiplex PCR

**Fig. 1:**
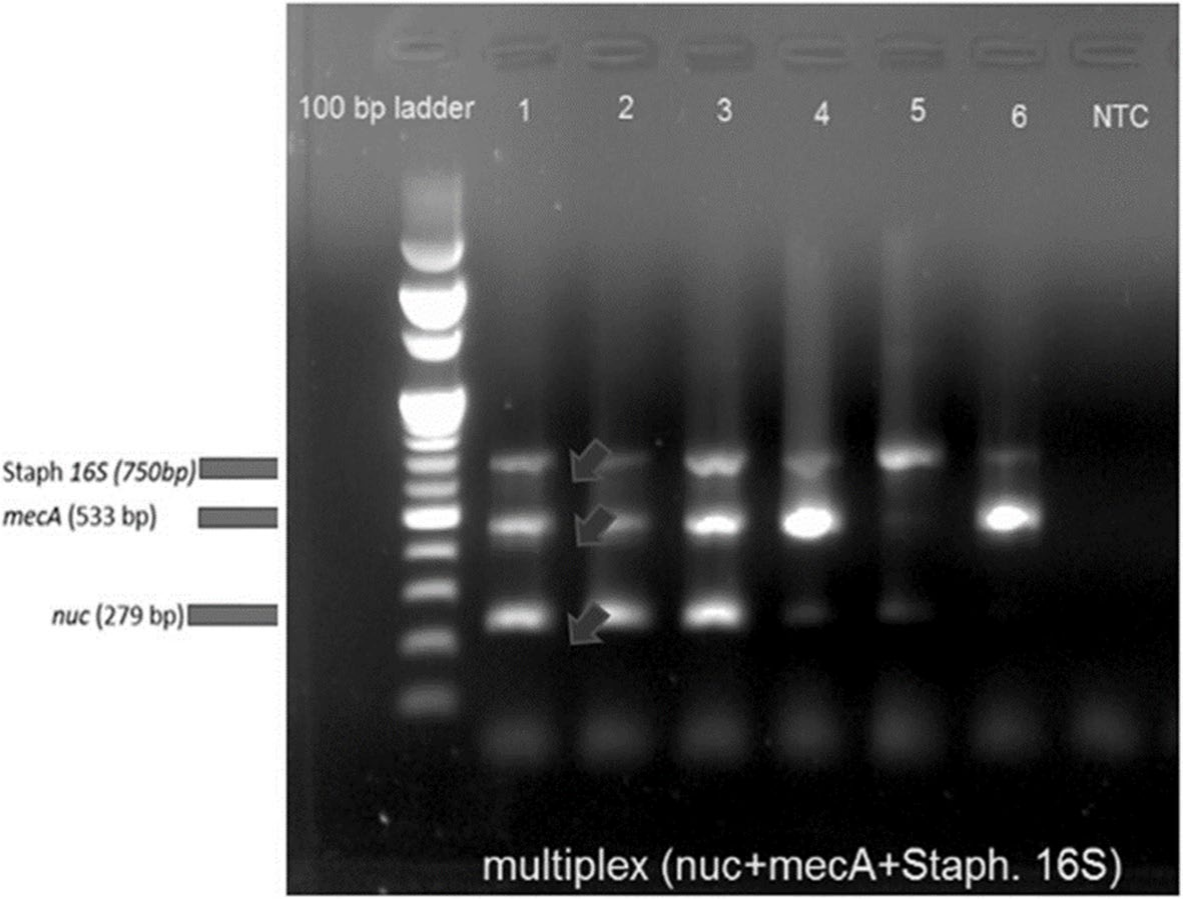
Multiplex PCR: For identification and detection of SA and MRSA, a multiplex PCR panel was developed. Sample 1 (positive control), NTC is negative control

Multiplex PCR based identification of MRSA from the cultured *S. aureus* isolates was performed. Standard DNA isolation method was used to isolate microbial DNA from culture [25]. 1 ml of cultured bacteria was centrifuged at 8000 g for 2 min and the supernatant was discarded. Cells were cleansed using STE buffer (Sodium Chloride-Tris–EDTA), centrifuged at 8000 g for 2 min and then the cells were resuspended in TE buffer. Tris-saturated phenol was mixed thoroughly and centrifuged at 13000 g for 5 min at 4 °C. Aqueous layer was taken in a fresh microcentrifuge tube (MCT) and chloroform was added, then centrifuged at 13000 g for 5 min at 4 °C. The aqueous layer containing the isolated DNA was collected in a fresh MCT and its purity and concentration were checked spectrophotometrically by using nanodrop. For identification of *Staphylococcus aureus* and methicillin resistant *Staphylococcus aureus*, Multiplex-PCR was standardized for 4 genes, viz. *Staphylococcus* genus specific 16S gene, *S. aureus* specific nuclease gene, methicillin resistance gene *PBP2A* (*mecA*) and MRSA virulence factor *PVL* gene (Table 1). *PVL* is a virulence factor of community-acquired MRSA (CA-MRSA) thathelps to distinguish from hospital acquired-MRSA. Primers were taken from previous studies [26–30] and checked for their specificity using NCBI primer blast [31]. In case of any discrepancy among the culture-based results and PCR based results, PCR based results were considered for further analysis (Fig. 1).

**Table 1:** Primer sequences and their amplicon length to detect SA and MRSA in culture isolates

Gene region	Primer Sequence (5’- 3’)	Amplicon Sequence
*nuc* gene	5’ GCGATTGATGGTGATACGGT 3’	279 bp
	5’ AGCCAAGCCTTGACGAACTAAAGC 3’	
*mecA* gene	5’ AAAATCGATGGTAAAGGTTGGC 3’	533 bp
	5’ AGTTCTGCAGTACCGGATTTGC 3’	
*16S* gene	5’ GTTATTAGGGAAGAACATATGTG 3’	750 bp
	5’ CCACCTTCCTCCGGTTTGTCACC 3’	
PVL gene	5’ ATCATTAGGTAAAATGTCTGGACATGATCCA 3’	410 bp
	5’ GCATCAAGTGTATTGGATAGCAAAAGC 3’

### Antibiotic sensitivity testing

All the wound MRSA isolates of D+F+ group were tested for sensitivity to the commonly used first- and second-line antibiotics by Kirby Bauer Disc diffusion method. MRSA isolates were tested for sensitivity to the following antibiotics as per CLSI guidelines: Penicillin (10 units), Cefoxitin (30 µg), Levofloxacin (5 µg), Cotrimoxazole (25 µg), Vancomycin (30 µg), Linezolid (30 µg), Clindamycin (2 µg), Azithromycin (15 µg), Tetracycline (30 µg) and Chloramphenicol (30 µg) [32]. Based on zone of inhibition results, isolates were categorized as susceptible/sensitive, intermediate (moderately susceptible/sensitive) and resistant to specific antibiotics [33]. MRSA isolates from wounds were identified as multidrug resistant (MDR) based on the standard definitions for acquired resistance guidelines [33].

### Statistical tests and data analysis

The study aimed to assess the carriage rates of *Staphylococcus aureus* (SA) and methicillin-resistant *Staphylococcus aureus* (MRSA) in nasal and foot skin sites among three groups (D+F+, D+F-, and D-F-). MRSA or SA carriage rate is defined as the percentage of individuals in a group that are positive for MRSA or SA. Analysis of variance (ANOVA) test was performed to examine the significant difference in carriage rates across the three groups. To check for the directionality of the association we have performed chi-square post hoc test of trend. Further for pairwise comparisons chi-square test was conducted to compare SA and MRSA carriage between the groups. In D+F+ group, Student’s T-test was done for comparing clinical parameters [HbA1C, Fasting blood glucose (FBG) and Postprandial blood glucose (PPBG)] between MRSA positive and MRSA negative subgroups of DFU wounds. Binary logistic regression was performed to check for the association of SA and MRSA presence between both nasal and wound site and subsequently odds ratio was estimated to quantify the strength of the association. Positive predictive value (PPV) and negative predictive values (NPV) were also estimated to determine the predictive accuracy of the regression model.

## Results

### Carriage of SA and MRSA

The mean age of the D+F+ patients is 51.67 ± 8.48 years and 63.3% of the patients are male. For D+F- and D-F- groups, the mean ages are 47.22 ± 8.97 years and 47.1 ± 13.67 years respectively and the proportion of males for both D+F- and D-F- individuals is 53.8%.

**Fig. 2:**
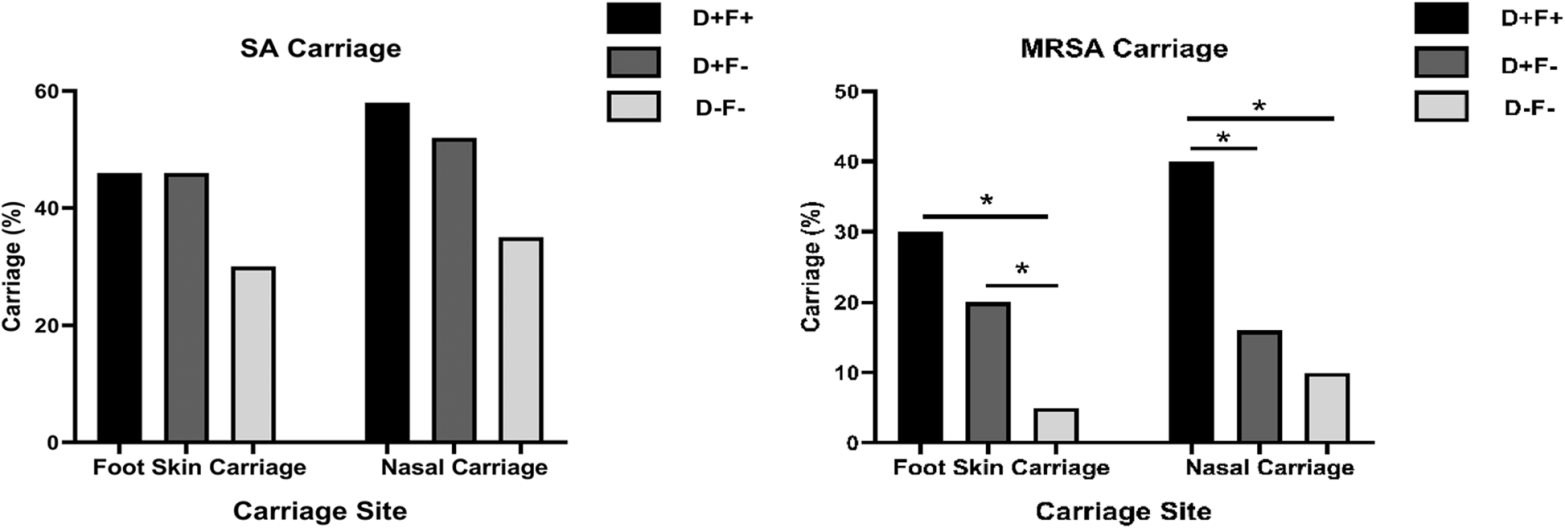
SA and MRSA carriage in nasal and the foot skin are compared among three groups. Nasal MRSA carriage of the D+F+ group (40%) was significantly higher compared to both the D+F- and D-F- control groups (16% and 10%, *p*-value < 0.05) (* means *p* < 0.05)

Carriage of SA and MRSA in nasal and foot skin sites were compared among three groups (D+F+ vs. D+F- vs. D-F-). Nasal and foot skin SA carriage of D+F+ (58% and 46%) and D+F- (52% and 46%) were moderately similar but was lower in the D-F- group (30% and 35%), although not significant (P_ANOVA_ > 0.05). SA carriage in the wound site was found to be 44%. (Table 2). These findings indicate that there is no substantial variation in presence of SA among these groups at foot skin sites and the nose (Fig. 2).

**Table 2:** SA carriage among three different groups (D+F+ ,D+F- and D-F-) on different body sites

Group	Sample size	FOOT -SA presence	NOSE-SA presence	Wound-SA Presence	Foot carriage	Nose carriage	Wound carriage
D+F+	50	23	29	22	46%	58%	44%
D+F-	50	23	26	-	46%	52%	-
D-F-	40	12	14	-	30%	35%	-

We have compared nasal and foot MRSA carriage among three groups (D+F+ vs. D+F- vs. D-F-) and observed a significant difference of MRSA in both foot skin sites (P_ANOVA_ = 0.01) and the nasal cavity (P_ANOVA_ = 0.002) across the three groups (Table 3). To investigate the directionality of MRSA carriage for both nose and foot skin from healthy to diabetes to DFU individuals we have performed chi square post hoc test of trend analysis. We have observed a significant trend of increase in nasal (10% < 16% < 40%, P_trend-chi_ = 0.0005) and foot (5% < 20% < 30%, P_trend-chi_ = 0.003) MRSA carriage from healthy to diabetes to DFU individuals. Pairwise comparison showed that the prevalence of nasal MRSA carriage was significantly higher in the D+F+ group (40%) compared to both the D+F- and D-F- groups (16% and 10% respectively, *p*-value < 0.05) but in the foot skin site the proportion of individuals with MRSA carriage was higher in the D+F+ group (30%) compared to the D+F- group (20%, *p* > 0.05) and significantly higher in the D-F- group (5%, *p*-value < 0.05). Thus, the observed gradual increase in MRSA carriage from the healthy group to diabetes to DFU individuals and the significant difference in nasal MRSA carriage by pairwise comparison of all the three groups highlights that the diabetes patients with or without foot ulcer may act as reservoirs of MRSA in nasal cavity.

**Table 3:** MRSA carriage among three different groups (D+F+, D+F- and D-F-) on different body sites

Group	Sample size	MRSA in foot	MRSA in Nose	MRSA in wound	Foot carriage	Nose carriage	Wound carriage
D+F+	50	15	20	14	30%	40%	28%
D+F-	50	10	8	-	20%	16%	-
D-F-	40	2	4	-	5%	10%	-

### Comparison of clinical parameters based on presence or absence of MRSA

**Fig. 3:**
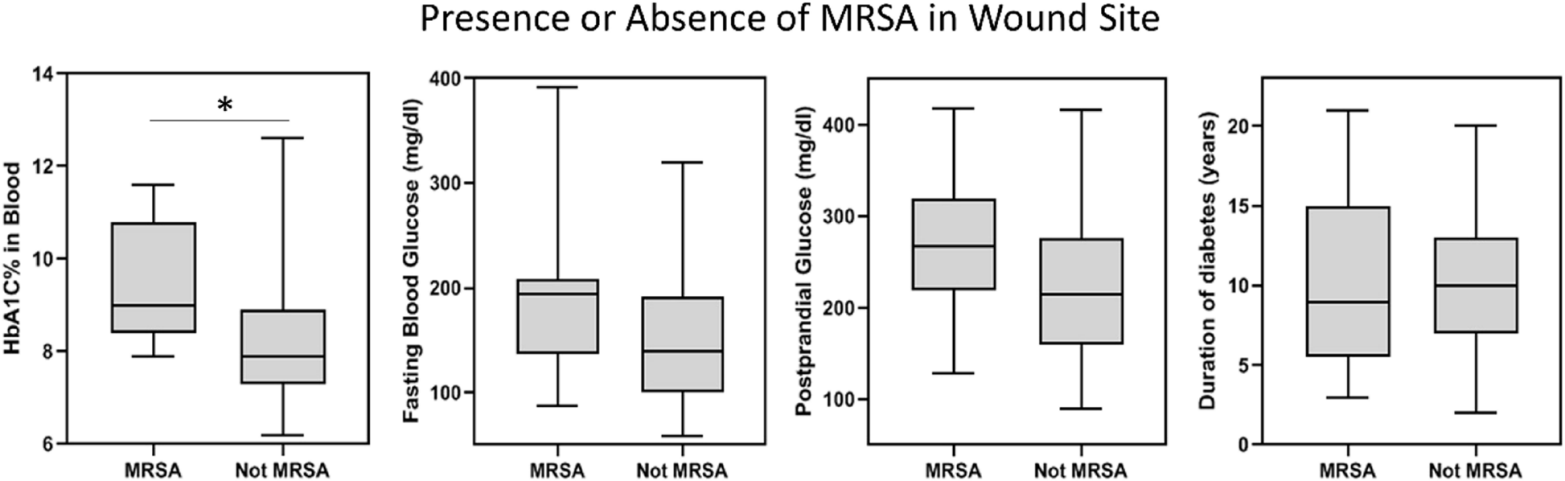
Comparison of clinical parameters. HbA1c, FBG, PPBG & duration of diabetes in D+F+ patients are compared between MRSA positive and MRSA negative group. HbA1C level is significantly higher (*p* < 0.02) in D+F+ patients with MRSA on their wound sites.)

Clinical parameters like HbA1C, fasting blood glucose (FBG) and Postprandial blood glucose (PPBG) were checked for association with MRSA carriage in all the three groups (D+F+, D+F- and D-F- patients). For this the D+F+ group is categorized based on the presence (MRSA+ ve) or absence (MRSA-ve) of MRSA and clinical parameters (i.e. HbA1C, FBG, PPBG & duration of diabetes) are compared between the two groups for nasal, wound and foot skin sites separately. The HbA1C level was found to be significantly higher (*p* < 0.02) in D+F+ patients with MRSA in their wound sites. Other group comparisons were not found to be statistically significant (Fig. 3).

### Nasal MRSA carriage is associated with wound MRSA carriage in DFU patients

In the D+F+ group (*n* = 50), SA and MRSA were identified in 22 patients (44%) and 14 patients (28%) respectively from wound swabs. Among the 22 patients with SA colonization in wound, 16 were nasal SA+ve and 11 were foot SA+ve. Also, of the 14 patients that have wound MRSA colonization, 9 were nasal MRSA + ve and 5 were foot skin site MRSA + ve. We have performed binary logistic regression and found that nasal MRSA colonization was an independent predictor for wound MRSA infection (OR = 4.09, 95%CI: 1.12 -15.05; *p* = 0.03), but nasal SA colonization was not an independent predictor for wound SA infection (OR = 3.07, 95% CI: 0.9 -10.18; *p* = 0.06) (Table 4). We have also observed 64% positive predictive value (PPV) and 69% negative predictive value (NPV) for presence and absence of MRSA in both nasal and wound site respectively.

**Table 4:** Corelation of nasal SA and MRSA with wound site SA and MRSA in D+F+ group

Characteristics	Ulcer SA + (*n* = 22)	Ulcer MRSA + (*n* = 14)	*p*-value	Odds ratio (95% CI)	sensitivity	specificity	NPV	PPV
Nasal SA+ (*n* = 29)	16	10	0.06	3.0769 (0.92- 10.18) for SA in ulcer	55.17%	71.43%	53.57%	72.73%
Nasal MRSA+ (*n* = 20)	11	9	0.03*	4.0909 (1.12 -15.05) for MRSA in ulcer	45%	83.33%	69.44%	64.29%

^*^is statistically significant

### Multiplex PCR designing

We have successfully developed a multiplex PCR assay for the identification of SA and MRSA. We have used four primers specific for16S,nuc, mecA,, Pvl gene region to identify *Staphylococcus* genus specific *16S* gene, *S. aureus* specific *nuclease* gene,methicillin resistance gene PBP2A (*mecA*) and community acquired MRSA infection detection gene Pvl. [26–30] The multiplex PCR has been validated for rapid detection of MRSA from microbial DNA directly isolated from wound swabs.

### Antibiotic sensitivity testing results

**Fig. 4:**
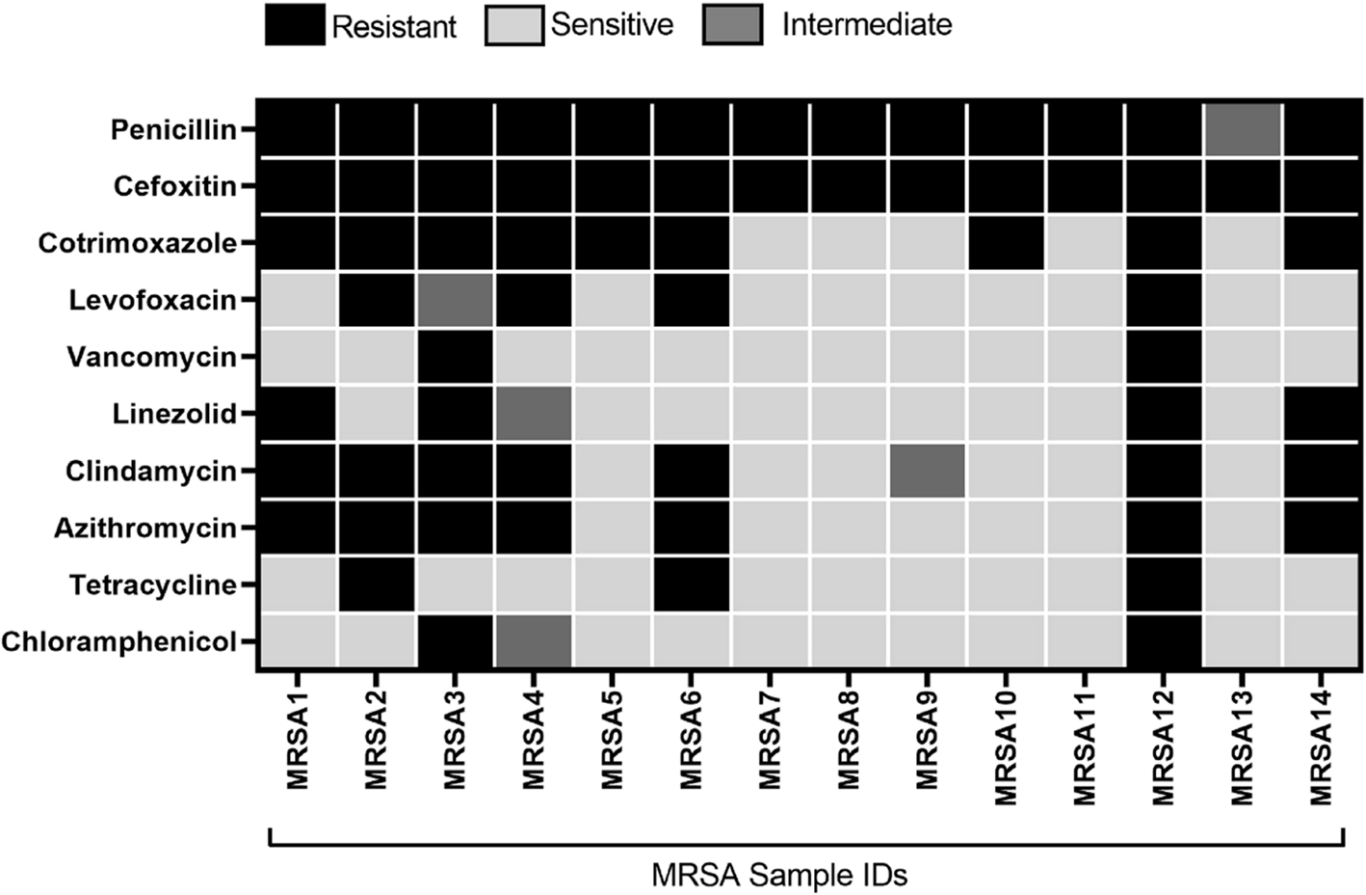
Heatmap of Antibiotic Sensitivity result: Antibiotic Sensitivity testing result shows that MRSA isolates from wound were mostly multidrug resistance (MDR) (*n* = 9/14, 64%) [MRSA1 – MRSA6, MRSA10, MRSA12, MRSA14]. Based on zone of inhibition results, isolates were categorized as susceptible/sensitive, intermediate (moderately susceptible/sensitive) and resistant to specific antibiotics

For the14 patients who have MRSA in their wound site, antibiotic sensitivity assays were performed. It was observed that majority of the MRSA isolates (*n* = 9/14, 64%) from wound were multidrug resistant (MDR) (Fig. 4). 92% of MRSA isolates were resistant to Penicillin and at least ~ 50% of the MRSA were resistant to Azithromycin, Clindamycin and Cotrimoxazole. However, most of the MRSA were sensitive to Chloramphenicol (11/14, 78%), Vancomycin (12/14, 85%) and tetracycline (11/14, 78%).

## Discussion

This study focused on the Eastern Indian Population and all the study participants were recruited from the dedicated Diabetic Foot Clinic (attendance 20 patients/week) under the diabetes outpatients services (footfall 800 patients/ week) of IPGMER Kolkata, India. The nasal MRSA carriage rate of DFU patients were 40% which is significantly higher (*p* < 0.05) than healthy controls (10%). The nasal MRSA carriage rate in DFU patients was also much higher in our study than USA (8.8%) [19], France (16.5%) [9], and Taiwan (5.4%) [34]. In D+F+ group, wound MRSA carriage was 28% and concordance between nasal and ulcer MRSA was 64% (9/14). For foot skin site, the D-F- had significantly lower MRSA carriage (5%) compared to other two groups (D+F+  = 30%, and D+F- = 20%). This suggest that colonization of MRSA in foot skin site is more likely to be as commensals but nasal MRSA can act as a risk factor for chronic diabetic foot ulcers [19, 34].

Previous studies also suggest association between nasal SA/MRSA colonization with chronic ulcer SA/MRSA colonization. Haleem *et. al* reported that 31.6% patients had nasal SA Carriage and 36.7% had wound SA carriage. However, only 8.8% subject had nasal MRSA colonies and 8.8% had wound MRSA [19]. In shin-yi Lin et al.study, diabetic patients with foot ulcer had nasal MRSA carriage of 5.4% and nasal MRSA colonization was independent predictor of wound MRSA infection. (OR: 19.09, 95% CI: 2.12–171.91) [34]. In the Indian population, we have also found nasal MRSA colonization was an independent predictor for wound MRSA infection in D+F+ group (odds ratio: 4.09, 95% CI: 1.12–15.05). It signifies that, nasal MRSA carriage in diabetic foot ulcer patients is a significant risk factor for wound site MRSA infection and these patients had fourfold increased risk than those without nasal MRSA colonization. Both the shin-yi Lin et al.study and our study raises concern about growing threat of nasal MRSA carriage in diabetic patients that can act as a potential risk factor for developing antibiotic resistance in non -healing DFU wounds globally.

We have further observed 64% positive predictive value (PPV) and 69% negative predictive value (NPV) for presence and absence of MRSA respectively in both nasal and wound site. DFU infection caused by MDR (multidrug resistant) bacteria were hard to treat and for long term infection, it can cause amputation of the leg [35]. In our study, most of the MRSA isolated from wound were MDR too. This suggests that detection of MRSA is vital for efficient disease prognosis New approaches are required to improve the management of Diabetic foot ulcer patients.

Different clinical parameters were also tested to check if the presence or absence of MRSA is influenced by them. Interestingly, no other body site carriage of any group except the wound of the DFU patients was significantly associated with any clinical data. HbA1C% (*p* < 0.05) was significantly higher in MRSA positive (in wound site) DFU patients compared to those who did not carry any MRSA in their wound site. This may be possible because MRSA or any bacteria relies on the nutrient source. In the case of the DFU site, bacteria have direct access to the blood nutrients, especially the presence of glycated hemoglobin that provides required nutrition to MRSA [36]. Also higher HbA1c level hampers phagocytic activity in the body, which may be the reason for MRSA colonization [37]. Another study has also shown an association between MRSA and high blood glucose levels [38]. Also, this suggests that controlling HbA1C can potentially be helpful for the treatment of infections in chronic DFUs. Our results strongly suggest that individuals with diabetes should be regularly screened for the presence of MRSA, which can be done by using PCR based platforms. For that reason, we have developed a rapid multiplex PCR based detection assay that has been used for identifying MRSA and SA from culture isolates.

In summary, our work provides evidence about the pattern of SA and MRSA colonization in infectious DFU patients and our designed multiplex PCR assay will be the easiest approach for the SA/MRSA identification in clinical environments. We hope to explore this study to a larger extent in future.

## Conclusions

Nasal MRSA carriage is significantly higher in diabetic foot ulcer patients than diabetic and non-diabetic individuals without foot ulcer. We have developed a Multiplex PCR assay for accurate detection of MRSA from culture isolates. Nasal MRSA colonization in DFU patient was an independent predictor for wound site MRSA infection thereby suggesting for earlier detection reducing the risk of wound MRSA infection. Further studies are needed to investigate whether decolonization of nasal MRSA in patients with diabetic foot ulcers can reduce the risk of wound site MRSA infection and improve clinical outcomes.

## Data Availability

All data generated or analysed during this study were included in this article.

## Abbreviations

DFU: Diabetic foot ulcer
SA: *Staphylococcus aureus*
MRSA: Methicillin-resistant *S. aureus*
O.R.: Odds Ratio
